# The beneficial effects of the composite probiotics from camel milk on glucose and lipid metabolism, liver and renal function and gut microbiota in *db/db* mice

**DOI:** 10.1186/s12906-021-03303-4

**Published:** 2021-04-22

**Authors:** Tabusi Manaer, Lan Yu, Xin-Hua Nabi, Dinareer Dilidaxi, Lu Liu, Jialehasibieke Sailike

**Affiliations:** 1 College of Pharmaceutical Sciences, Xinjiang Medical University, Urumqi, 830011 China; 2 Xinjiang Uygur Autonomous Region Institute for Drug Control, Urumqi, 830054 China; 3 The First Affiliated Hospital of Xinjiang Medical University, Urumqi, 830011 China

**Keywords:** Composite probiotics camel milk, *db/db* mice, Gut microbiota, TFCW, TG

## Abstract

**Background:**

Probiotics may have beneficial effects on patients with type 2 diabetes mellitus (T2DM). We separated 4 lactobacillus and 1 saccharomycetes from traditional fermented cheese whey (TFCW) and prepared composite probiotics from camel milk (CPCM) and investigated their effects on glucose and lipid metabolism, liver and renal function and gut microbiota in *db/db* mice.

**Methods:**

CPCM was prepared in the laboratory and 40 *db/db* mice were randomly divided into 4 groups as metformin, low-dose and high-dose group and model group, and treated for 6 weeks. In addition, 10 C57BL/Ks mice as normal control group were used for comparison. Fasting blood glucose (FBG), body weight (BW), oral glucose tolerance test (OGTT), glycated hemoglobin (HbAlc), C-peptide (CP), triglycerides (TG), total cholesterol (TC), low-density lipoprotein cholesterol (LDL-C), high density lipoprotein cholesterol (HDL-C), 24 h urinary microalbumin (24 h malb), urine ketone, urine sugar, pancreas and liver tissue and intestinal flora were tested.

**Results:**

Compared to diabetic group, high dose CPCM significantly decreased FBG, OGTT, HbAlc and IRI, plasma TC, TG, LDL-C, 24 h malb, urine ketone and urine sugar, increased CP, HDL-C levels, improved the liver and kidney function, protected the function of islets, also increased intestinal tract lactic acid bacteria and *Bifidobacterium*, decreased *Escherichia* in *db/db* mice.

**Conclusion:**

CPCM decreased FBG, OGTT and HbAlc, increased CP, modulated lipid metabolism and improved liver and kidney protected injury in *db/db* mice, which may be related to various probiotics acting through protecting the function of islets and regulating intestinal flora disturbance.

**Supplementary Information:**

The online version contains supplementary material available at 10.1186/s12906-021-03303-4.

## Background

Diabetes mellitus (DM) is the most prevalent disease of endocrinology and metabolism world wide and in China. DM prevalence in general population aged 20–79 years is 8.3% by 2013, with an increasing trend, and 80% of them is occuring in developing countries. In China, DM is affecting 11.6% of the population (approximately 1.14 hundred million) and 50.1% (approximately 4.93 hundred million) of population is in pre-diabetic condition [1, 2]. According to the International Diabetes Federation (IDF), in the year 2019, approximately 463 million adults worldwide were living with diabetes [3]. The total prevalence of diabetes is increasing and is expected to be 700 million by 2045 [3]. Treatment of DM encompasses insulin and anti-diabetic agents, whereas they have some side effects such as hypoglycemia, and injury to liver and renal function [4]. Recent studies have found that, prevalence of DM and impaired fasting blood glucose in Kazakh ethnic groups living in Northwest China is significantly lower than that of other ethnic groups there [5–9]. The main difference of Kazakh with other ethnic groups in this aspect is that, they use fermented dairy products in their life extensively as beverages and as traditional medicine for tuberculosis and adjuvant therapy of DM and traditional fermented cheese whey (TFCW) for regulation of lipid metabolism [10–12].

Fermented dairy products are rich in microorganisms, specially in lactobacillus and saccharomycetes, which are the main probiotics that play significant roles mentioned above [10–12]. Probiotics are mainly the lactic acid bacteria, including *lactobacillus, galactococcus, bifidobacterium* and *streptococcus*, which contain the roles of improving glucose and lipid metabolism, and alleviating constipation and diarrhea [13]. Animal studies also reported similar results that, probiotics lower blood glucose, and blood pressure [14]. Structural changes in gut microbiota are involved in the progression and development of T2DM, evidenced as decreased number of Firmicutes and increased number of Fusiformis in the gut of patients with T2DM [15]. *L.kefiranofaciens* is the most common probiotics in the fermented dairy products and its product, Kefiran, is evidenced to have anti-inflammatory roles [16] and to activate innate immunity without side effects [17]. *L.helveticus* elevates SIgA levels in gut mucosa and serum IgG levels and improves the phagocytic ability of the macrophagocyte [18]. *Lactococcus lactis* present heterologous proteins to the mucosal immune system in an effective way and induce specific immune response and its product, interleukin (IL) -12, can treat intestinal disease [19]. *L.plantarum* as the one of the important probiotics, maintains the balance of gut microbiota, elevates immunity, stimulates the macrophagocyte to secrete IL-6 and tumor necrosis factor-alpha and motivates the synthesis of IL-10, − 4, − 5 and − 1 to facilitate proliferation of lymphocytes and their participation in the activation of T cells, CTL, NK and LAK cells [20]. *L.plantarum* also can regulate the lipid and glucose metabolism [20]. *I.orientalis* is an important saccharomycetes and mainly used for the fermentation of wine [21].

In our previous work, we found that TFCW has anti-atherosclerotic and anti-inflammatory roles in rats through PPARγ and NF-kB pathways [10] and we identified the probiotics in TFCW which has anti-atherosclerotic effects in rabbits [11] and also identified one lactic acid bacteria in Xinjiang traditional fermented camel milk [22] as well as found that probiotic fermented camel milk has significant hypoglycemic potential in rats models of type 2 diabetes mellitus (T2DM) and may modulate lipid metabolism and protect renal function in the condition of T2DM, which might be related to various probiotics acting through promoting the release of GLP-1 and improving the function of β-cells [23], These probiotics have beneficial characteristics, including acid resistance, bile tolerance, high self-aggregation ability, high adhesion to Caco-2 cells and so on [24, 25]. In our the latest work, team isolated 14 probiotics from fermented camel milk and found that the composite probiotics increased SCFA-producing bacteria and decreased *Escherichia coli*, promoted GLP-1 secretion by upregulation of GPR43/41, GCG and PC1/3 activity and inhibited apoptosis via regulating PI3K/AKT signaling pathways. The anti-diabetic effect of 14 probiotics in *db/db* mice seem to be related to an increase of SCFA-producing bacteria, the improvement of intestinal barrier function and the upregulation of GLP-1 production [26].

There is increasing evidence showing that the relationship between gut microbiota and diabetes is intertwined. Gut microorganisms are part of an extremely sophisticated ecosystem. There are abundant, multifarious microorganisms living in the intestines, such as *Fusobacteria*, *Actinobacteria*, *Firmicutes*, and *Bacteroidetes*. Recent studies have shown that the composition and abundance of gut microbiota in healthy individuals are remarkably different from patients with T2D [27].

Therefore, it is reasonable to speculate that probiotics from TFCW may have beneficial effects in the *db/db* mice. So in this study, we separated 4 lactobacillus including *L.kefiranofaciens, L.plantarum, L.helveticus, L.lactis* and 1 saccharomycetes, *I.orientalis* from TFCW, identified at China Center of Industrial Culture Collection (CICC) and prepared CPCM in the laboratory and explored their effects on glucose and lipid metabolism, liver and renal function and gut microbiota in *db/db* mice.

### Objectives

The object of this study aimed to explore the possible anti-diabetic effects of 4 lactobacillus and 1 saccharomycetes from TFCW in *db/db* mice and aimed to prepare an effective CPCM to prevent and treat diabetes.

## Methods

### Materials

*Lactobacillus kefiranofaciens, Lactobacillus plantarum, Lactobacillus helveticus, Lactococcus lactis* and *Issatchenkia orientalis* were separated from TFCW and identified and stored at China Center of Industrial Culture Collection (CICC) until use. Camel milk was purchased from Jemsar county Xinjiang China. Man Rogosa Sharpe(MRS) broth, malt extract broth, MRS medium, sabouraud dextrose medium, anaerobic agar and potato dextrose agar were purchased from Qingdao Rishui Bio-Technologies Co., Ltd. with cat. Number: 11307, 11,147, 11,304, 11,196, 11,034 and 11,165 respectively. Metformin was purchased from Shanghai shiguibao pharmaceutical co. LTD (0.5 g, batch number: 1401087). Pentobarbital sodium was purchased from Merck & Co.(5 g, batch number: 1127H037). ELISA test kits for HbA1c, C-peptide, triglyceride, total cholesterol, low and high density lipoprotein cholesterol, and oral glucose tolerance test were purchased from Wuhan Huamei Bio-Technologies Co., Ltd. with batch number: C0519620165, Z29019698, M0519620161, M0519620162, M0519620163, M0519620164 and D0519620151 respectively.

### Ethical statement

All animal care and experimental procedures were approved by the ethics committee for animal study of the First Affiliated Hospital of Xinjiang Medical University (IACUC-20140716009).

### Study design and allocating animals to experimental groups

6 week old *db/db* mice (*n* = 40, female:male = 1:1) and C57BL/Ks mice (*n* = 10 female:male = 1:1) using the specific pathogen free (SPF) grading were purchased from Changzhou cavins experimental animal co. LTD (SCXK 2011–0003) [28]. *db/db* mice were randomly divided into 4 group (*n* = 10 for each group and male:female = 1:1) by blood glucose and weight as model group, metformin group, and low and high dose CPCM groups after 1 week adaptation. The 10 C57BL/Ks mice (the same aged, male:female = 1:1) were selected as normal group. *db/db* mice were randomly divided into 4 group (*n* = 10 for each group and male:female = 1:1) by blood glucose and weight as model group, metformin group, and low and high dose CPCM groups after 1 week adaptation. The 10 C57BL/Ks mice (the same aged, male:female = 1:1) were selected as normal group.

### Experimental procedures

All the mice had free access to food and water and at 09:00 am each day normal and model groups were given disinfected-skimmed camel milk, metformin group was given metformin of 0.3 g per 1 kg body weight and low and high dose probiotic groups were given low and high dose CPCM. Appearance, food and water intake, and environment of study animals were observed everyday; weight and fasting blood glucose were measured once a week. At the last week, OGTT was performed after fasting 12 h by giving glucose 2 g per kg body weight and measuring blood glucose at 0, 30, 60, and 120 min and calculating AUC. 24 h urine sample was collected at the last day of the experiment, centrifuged at 3000 rpm/min for 10 min and 2 ml sample was used for 24 h mALB measurement. At the 6th week, animals were fasted 12 h, and anesthetized with an intraperitoneal injection with a dose of 30 mg·kg^− 1^ of 3% pentobarbital sodium solution(Merck & Co.,), blood was put in EDTA tube and centrifuged after blood sampling and plasma was stored at − 20 °C. HbA1c, C-peptide, triglyceride, total cholesterol, and high and low density lipoprotein cholesterol were measured. Liver, pancreas and kidney were collected, weighed, fixed in paraformaldehyde solution and pathological section was made, IRI was calculated using IRI = 1.5 + FBG × fasting CP (ng·mL^− 1^ × 333)/2800 and compared IRI at the 6th week among groups. Colon and its contents were collected and stored in bio-bag at − 80 °C. Histological analysis were performed by way of optical microscopy on paraffin material of the pancreas and liver. Pancreas and liver tissue sections were fixed in 10% buffered formalin and immediately histological preparations were made. An amount of 5 μm thick sections were cut and stained with haematoxylene and eosin (HE) for histological analysis.

### Housing and husbandry

*db/db* mice and C57BL/Ks mice using SPF grading were purchased from Changzhou cavins experimental animal co. LTD. Study animals were kept in temperature 23 ± 1 °C, moisture 50 ± 5% and 12 h light. All the mice were free for food and water and at 09:00 am each day normal and model groups were given disinfected-skimmed camel milk, metformin group was given metformin of 0.3 g per 1 kg body weight and low and high dose probiotic groups were given low and high dose CPCM. Appearance, food and water intake, and environment of study animals were observed everyday.

### Methods of euthanasia/sacrifice

At the 6th week (At the end of the 42 days), animals were fasted 12 h, and anesthetized with an intraperitoneal injection with injection dosages of 30 mg·kg^− 1^ of 3% pentobarbital sodium solution (manufacturer: Merck & Co., 5 g, batch number: 1127H037). After loss of consciousness, blood samples were collected from the abdominal aorta and the mice were sacrificed by cervical dislocation. The experimental protocol complied with the ARRIVE guidelines and was carried in compliance with National Institutes of Health guide for the care and use of Laboratory animals. All animal care and experimental procedures were approved by the ethics committee for animal study of the First Affiliated Hospital of Xinjiang Medical University (IACUC-20140716009).

### Preparation of the probiotic complex

Fresh camel milk was skimmed at 300 rpm/min and upper fat part was discarded 15-20Mpa for 1-2 min, disinfected on 95 °C for 10 min and stored at 40 °C until use. Stored Lactobacillus was selected and put in MRS broth at 37 °C for 24 h and activated until 2–3 generations for use. *I.orientalis* was selected and put in sabouraud dextrose medium and underlined, cultured at 37 °C for 48-72 h and a bacterial strain was put in malt extract broth to be cultured at 35 °C for 24 h and activated until 2–3 generations for use. Lactobacillus and *I.orientalis* prepared as above were centrifugated at 3000 rpm/min for 10 min, washed twice using 0.9%normal saline, collected and stored until use. Lactobacillus and *l.orientalis* were prepared and activated as above and were inoculated in fresh skimmed camel milk, cultured at 30 °C for 24 h and sampled each 3 h to measure calculate the viable count and pH. The viable count of the activated lactic acid bacteria and *I.orientalis* was tested, and then the four kinds of lactic acid bacteria were inoculated in sterile (95 °C, 10 min) volume ratio of 11% of freshly-defatted camel’s milk, the initial concentration of about 7.1 viable count, each of the lactic acid bacteria was inoculated in the same manner in two portions each, one part fermented separately, and other part was inoculated with *I.orientalis* having an initial logarithmic value of about 5.5 viable count. Each sample was fermented in a thermostat incubator at 30 °C for 12 h, and samples were taken periodically as required to determine the viable count was after pH was measured along with was after total acid content. pH measured by pH meter and total acid measured by according to GB 5413.34–2010 the method of determination of fermented milk acidity. Determination of viable count of lactobacillus bacteria count: the fermented milk diluted with a sterile gradient, take the appropriate dilution of fermented milk, MRS medium pouring culture, each dilution of the MRS medium to do two parallel samples in a 37 °C incubator anaerobic culture after 2–3 days count. Determination of viable count of *I.orientalis*: the fermented milk was diluted with aseptic gradient, fermented milk of appropriate dilution was selected and coated with potato dextrose agar. The culture MRS medium of each dilution was subjected to two parallel samples 30 °C incubator aerobic culture 2–3 days after counting. Preparation of CPCM: four kinds of lactic acid bacteria were suspended in the sterilized skim camel milk and the viable count of each lactic acid bacterium was adjusted to 1.0 × 10^8^ CFU/mL and 1.0 × 10^10^ CFU/mL, respectively. Viable count was determined with MRS medium before use. At the same time, *I.orientalis* were suspended in sterilized skim camel milk and their viable count was adjusted to 1.0 × 10^6^ CFU/mL and 1.0 × 10^8^ CFU/mL, respectively. Potato dextrose agar was used for viable count and the lactic acid bacteria and *I.orientalis* were prepared in the form of viable probiotics compound camel milk, adjust the pH was 7.0, including the low-dose group, the number of viable count of per lactic acid bacteria was 1.0 × 10^8^ CFU/mL and *I.orientalis* was 1.0 × 10^6^ CFU/mL and the high-dose group, the number of viable count of per lactic acid bacteria was 1.0 × 10^10^ CFU/mL and *I.orientalis* was 1.0 × 10^8^ CFU/mL, which were stored in frozen bottles according to the daily usage dose at − 80 °C for future use.

### Extraction of total bacterial DNA

Extraction of total bacterial DNA from the colon contents in *db/db* mice was performed according to the instructions of the QIAamp Fast DNA Stool Mini Kit (Qiagen, Germany). Specific steps are as follows: 1. 180-220 mg stool was weighed and put in 2 ml microcentrifuging tube and place tube on ice. 2. 1 ml inhibit EX Buffer was added to each stool sample and whirled for 1 min or until thoroughly homogenized. 3. Heat the suspension for 5 min at 70 °C. The lysis temperature can be increased to 95 °C for cells that are difficult to lyse for 15 s. 4. Samples were centrifuged for at 12000 rpm for 1 min to pellet stool particles. 5. 15ul proteinase K was added into a new 2 ml microcentrifuge tube. 6. 200ul supernatant sample from step 4 was put into the centrifuging tube of step 5. 7. 200ul Buffer AL was added and whirled for 15 s. 8. Samples were incubated at 70 °C for 10 min. 9. 200ul of ethanol was added to the lysate, and mixed by vortexing. 10. 600ul lysate from step 9 was carefully added into the QlAmp spin column, centrifuged at 12000 rpm for 1 min and put in a new 2 ml collection tube, and the tube containing the filtrate was discarded. 11. QIAamp spin column was carefully opened, 500ul Buffer AW1 was added, centrifuged at 12000 rpm for 1 min, and put in a new 2 ml collection tube, and the collection tube containing the filtrate was discarded. 12. The QIAamp spin column was carefully opened, 500ul Buffer AW2 was added and centrifuged for 3 min. 13. The QIAamp spin column was placed in a new 2 ml collection tube, the collection tube with filtrate was discarded and centrifuged for 3 min. 14.The QIAamp spin column was transferred into a new labeled 1.5 ml collection tube, the column was opened, 200ul BufferATE was added directly onto the QLAamp membrane, incubated at 25 °C for 1 min and then centrifuged for 1 min to elute DNA. Measurement of purity and concentration of bacterial total DNA in mouse feces: Electrophoresis identification, 5 ul of total bacterial DNA was mixed with 2ul of 10 × loading buffer and loaded on 1% agarose gel well, and it was first run at 100 V for 5 min and then run at 80 V for 50 min. After the electrophoresis was completed, the gel imager was used to image and observe the results. The purity and intactness of total bacterial DNA were determined according to the result of electrophoresis. Detection of nucleic acid protein analyzer identification, 2ul bacterial total DNA extract was put on NanoDrop1000 spectrophotometer to measure the total bacterial DNA absorbance at 260 nm and 280 nm, while AE liquid as the total bacterial DNA extract control, bacterial total DNA concentration and OD260/OD280 The ratio.

### Common PCR reaction system and the reaction program

Common PCR reaction system and the reaction program for *L.kefiranofaciens, L.plantarum, L.helveticus, L.lactis, Escherichia* and *Bifidobacterium* are as follows: Reaction system (20ul): 0.5ul upstream primer, Downstream primer 0.5ul, 2 × PCR Master Mix 10ul, DNA template 2ul, Nuclease-Free-water Replenishment to 20ul. Reaction procedure: Pre-denaturation: at 94 °C for 3 min, Denaturation: at 94 °C for 30s, Annealing: at 60 °C for 30s, Extension at 72 °C for 30s. Each sample was analysed in duplicate for 40 cycles. Final extension at 72 °C for 5 min, then at 4 °C Forever.

### Detection of 16S rDNA

Detection of 16S rDNA of *L.rhamnosus, L.lactis, L.helveticus, L.plantarum, Escherichia* and *Bifidobacterium* by RT-qPCR. Standard Preparation: *L.kefiranofaciens, L.plantarum, L.helveticus, L.lactis, Escherichia and Bifidobacterium* genus 16SrDNA variable region primers for PCR amplification products were obtained by electrophoresis amplified target fragment, and four kinds of lactic acid bacteria, Escherichia and Bifidobacterium genomic target gel cut, recovered as a DNA standard, according to the instructions of the TIANgel Midi Purification Kit (BEIJING TIANGEN BIOTECH CO., LTD, with cat. Number: DP209). the specific steps are as follows: Under a UV lamp, a single DNA band of interest was cut from the agarose gel with a razor and bladed into a new 1.5 mL centrifuge tube and weigh the net weight. An equal volume of PC solution was added to the centrifuge tube (add 100ul of PC solution to each 0.1 g gel), allowed to soak in a water bath at 50 °C for 10 min and the microcentrifuge tube was gently inverted every 2 ~ 3 min until the gel was completely dissolved. The solution was pipetted from the previous step, added into an adsorption column (the adsorption column is equilibrated before the experiment), centrifuged for 1 min at full speed to drain the waste in the collecting pipe, and put the column in the collecting pipe. 600ul PW rinsing solution was added to the column, centrifuged at full speed for 1 min, the waste liquid in the collecting tube was discarded, and the column was placed in a collecting tube. The step 4 was repeated. The column was put into the collection tube, centrifuged at full speed 2 min, the rinse was discarded, and the adsorption column was placed in 25 °C for a few minutes to be dried completely. The column was placed in a new centrifuge tube in the middle of the adsorption membrane, 50ul elution buffer EB was dropped, put at 25 °C for 2 min, centrifuged at full speed 2 min, and DNA solution was collected. Establishment of standard curve: *L.kefiranofaciens, L.plantarum, L.helveticus, L.lactis, Escherichia* and *Bifidobacterium* were respectively taken as a positive template and DNA samples with a concentration of 10^1^-10^8^copies/uL were sequentially gradient-diluted as a positive template. Nuclease-Free-water as a negative control, each sample was repeated in parallel 3 times, according to the following reaction system and reaction conditions, RT-qPCR reaction. ① reaction system (20uL): 0.5ul upstream primer, Downstream primer 0.5ul, Fluorescent dye SYBR GreenI 10ul, Template 2ul, Nuclease-Free-water Replenishment to 20ul. ② reaction conditions: Stage 1: Pre-denaturation at 50 °C for 2 min, at 95 °C for 10 min, 1 Cycle. Stage 2: PCR reaction at 95 °C for 15 s, at Tm for 45 s, at 72 °C for 30s, 40 Cycles. Stage 3: Dissociation Curve. Real-time fluorescent quantitative PCR thermal cycler (USA ABI Applied Biosystems 7500 Fast System) automatically generated amplification curve, and melting curve, and the product was confirmed by the melting curve. After the reaction was completed, the software attached to the instrument was used for analysis and a standard curve was automatically generated. The total bacterial DNA extract of colon from each group was analyzed according to the above reaction system, and the reaction conditions were RT -qPCR reaction. After the reaction was completed, the data were analyzed by Bio-rad IQ5 System software and the number of genus of *L.kefiranofaciens, L.plantarum, L.helveticus, L.lactis, Escherichia* and *Bifidobacterium.*

### Statistical methods

Data were expressed as means ± SEM if normally distributed and as median (P25, P75) if not. Distributions of each variable studied were first tested for normality and homogeneity using Levene test. Nonparametric test was applied for comparisons among groups. Statistical significance was determined by level of 0.05 on two-sided tests. The statistic analyses were performed using the program SPSS (version 18.0).

## Results

### The effects of CPCM on body weight (BW) and fasting blood glucose (FBG) in *db/db* mice

**Fig. 1:**
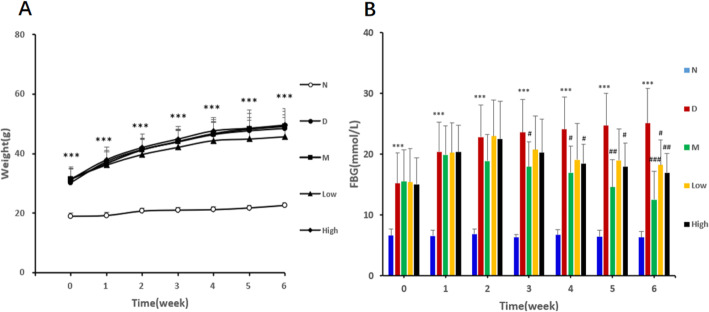
Effects of CPCM on body weight and Fasting blood glucose in *db/db* mice. ^***^P < 0.001 VS normal group; ^#^ *P* < 0.05, ^##^ *P* < 0.01 and ^###^ *P* < 0.001 VS model group. **a**. Body weight (g); **b**. Fasting blood glucose (mmol/L)

As given in Table 1 and Fig. 1, the effects of CPCM on body weight (BW) and fasting blood glucose (FBG) in *db/db* mice were observed before and at the 1st to 6th weeks of probiotic intervention. At baseline, normal group showed the lowest BW, compared with other groups. Model, metformin and low and high dose probiotic groups showed similar BW at the baseline. During the experiment process of 6 weeks, all the groups showed increasing BW, with the marked increase in model group. At the baseline, model group showed significantly higher FBG than did normal group (15.21 ± 5.07 vs 6.63 ± 1.04 mmol/L, *P* < 0.001), whereas there were no significant difference among other groups. During the experimental process, FBG in the model group showed significant consistent increasing trend with the highest at 6th week, compared to the baseline (25.11 ± 5.71 vs 15.21 ± 5.07 mmol/L, *P* < 0.001). FBG in the metformin group showed decreasing trend and it was significantly lower than the model group at the 3rd (17.92 ± 4.12 vs 23.58 ± 5.42 mmol/L, *P* < 0.05), 4th (16.99 ± 4.33 vs 24.09 ± 5.35 mmol/L, P < 0.05), 5th (14.62 ± 4.49 vs 24.70 ± 5.36 mmol/L, *P* < 0.01) and 6th week (12.51 ± 4.72 vs 25.11 ± 5.71 mmol/L, *P* < 0.01). FBG in the low dose probiotic group showed increasing trend from the baseline to the 3rd week, after that showed a decreasing trend and it was significantly lower than that of model group at the 6th week (18.22 ± 4.11 vs 25.11 ± 5.71 mmol/L, *P* < 0.05). FBG in the high dose probiotic group showed increasing trend from the baseline to the 3rd week, after that showed a decreasing trend and it was significantly lower than that of model group at the 4th (18.50 ± 3.10 vs 24.09 ± 5.35 mmol/L, *P* < 0.05), 5th (17.93 ± 3.90 vs 24.70 ± 5.36 mmol/L, *P* < 0.05) and 6th week (16.95 ± 3.18 vs 25.11 ± 5.71 mmol/L, *P* < 0.01).

**Table 1:** Effects of CPCM on body weight and Fasting blood glucose in *db/db* mice

	Normal group	Model group	Metformin group	Low dose group	High dose group
Body weight (g)					
Baseline	18.99±2.49	30.29±4.70^***^	31.36±4.22	31.62±3.16	31.33±3.69
1^st^ week	19.22±2.58	37.39±4.76^***^	36.68±3.83	36.17±4.32	37.90±3.05
2^nd^ week	20.68±3.07	41.43±5.25^***^	41.25±3.59	39.70±5.18	41.97±3.31
3^rd^ week	20.97±3.30	43.96±5.23^***^	43.99±3.66	42.10±5.96	44.79±3.45
4^th^ week	21.12±3.77	46.45±5.74^***^	46.67±3.85	44.39±6.31	47.59±3.90
5^th^ week	21.69±3.36	47.74±6.85^***^	48.22±3.87	44.89±6.40	48.44±5.18
6^th^ week	22.52±2.95	48.50±6.68^***^	49.08±3.93	45.68±6.50	49.51±4.65
Fasting blood glucose (mmol/L)					
Baseline	6.63±1.04	15.21±5.07^***^	15.51±5.26	15.41±5.53	15.03±4.40
1^st^ week	6.54±0.96	20.40±4.92 ^***^	19.91±4.77	20.23±4.94	20.36±4.46
2^nd^ week	6.87±0.80	22.84±5.29^***^	18.88±4.40	22.98±5.98	22.49±6.27
3^rd^ week	6.33±0.51	23.58±5.42^***^	17.92±4.12^#^	20.74±5.59	20.25±5.55
4^th^ week	6.72±0.91	24.09±5.35^***^	16.99±4.33^#^	19.11±5.96	18.50±3.10^#^
5^th^ week	6.45±1.08	24.70±5.36^***^	14.62±4.49^##^	18.98±5.16	17.93±3.90^#^
6^th^ week	6.31±0.96	25.11±5.71^***^	12.51±4.72^###^	18.22±4.11^#^	16.95±3.18^##^

^***^P <0.001 VS normal group; ^#^ *P* <0.05, ^##^ *P* <0.01 and ^###^ *P* <0.001 VS model group

### The effects of CPCM on oral glucose tolerance test (OGTT), glycosylated hemoglobin (HbA1c), C-peptide (CP) and insulin resistance index (IRI) in *db/db* mice

**Fig. 2:**
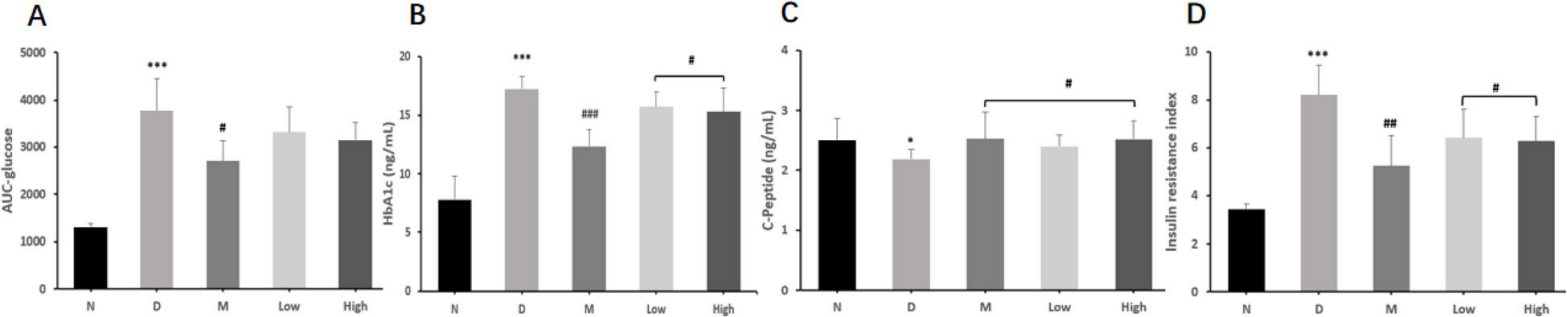
Effect of CPCM on AUC, glycosylated hemoglobin, C-peptide and insulin resistance index in *db/db* mice. ^*^P < 0.05, ^***^P < 0.001 vs normal group; ^#^ *P* < 0.05, ^##^ *P* < 0.01, and ^*###*^ *P* < 0.001 vs model group. **a**. AUC; **b**. glycosylated hemoglobin (ng/ml); **c**. C-peptide (mmol/L); **d**. insulin resistance index

As displayed in Table 2 and Fig. 2, the effects of CPCM on oral glucose tolerance test (OGTT), glycosylated hemoglobin (HbA1c), C-peptide (CP) and insulin resistance index (IRI) in *db/db* mice were observed at the 6th week of experiment. Model group showed significantly higher OGTT results at 0 (25.88 ± 6.19 vs 6.69 ± 0.71 mmol/L, *P* < 0.001), 30 (39.11 ± 4.34 vs 15.70 ± 2.50 mmol/L, *P* < 0.001), 60 (36.19 ± 8.55 vs 11.98 ± 1.86 mmol/L, *P* < 0.001) and 120 min (26.59 ± 6.52 vs 7.65 ± 2.35 mmol/L, *P* < 0.01) than did normal group. Compared with the model group, metformin group showed significantly lower OGTT results at 0 (13.10 ± 3.86 vs 25.88 ± 6.19 mmol/L, *P* < 0.001), 30 (31.51 ± 6.57 vs 39.11 ± 4.34 mmol/L, *P* < 0.05), 60 (24.53 ± 4.49 vs 36.19 ± 8.55, *P* < 0.05) and 120 min (16.19 ± 4.52 vs 26.59 ± 6.52 mmol/L, *P* < 0.01). Low dose probiotic group at 60 min (19.41 ± 3. 77 vs 26.59 ± 6.52 mmol/L, *P* < 0.05) and high dose probiotic groups at 0 (18.03 ± 4.46 vs 25.88 ± 6.19 mmol/L, *P* < 0.05), 60 (27.47 ± 4.12 vs 36.19 ± 8.55 mmol/L, *P* < 0.05) and 120 min (18.15 ± 4.49 vs 26.59 ± 6.52 mmol/L, *P* < 0.05) showed significantly lower OGTT results than did model group and the effects of probiotics on OGTT showed a dose-dependent characteristic. At the 6th week, model group showed the highest HbA1c than did any other groups; metformin (12.33 ± 1.44 vs 17.27 ± 1.01 ng/mL, *P* < 0.001), and low (15.74 ± 1.27 vs 17.27 ± 1.01 ng/mL, *P* < 0.05) and high dose (15.28 ± 2.04 vs 17.27 ± 1.01 ng/mL, *P* < 0.05) probiotic groups showed significantly lower HbA1c levels than did model group. At the 6th week, model group showed the lowest CP than did any other groups; metformin (2.53 ± 0.45 vs 2.19 ± 0.16 mmol/L, *P* < 0.01), and low (2.40 ± 0.19 vs 2.19 ± 0.16 mmol/L, *P* < 0.05) and high dose (2.52 ± 0.31 vs 2.19 ± 0.16 mmol/L, *P* < 0.05) probiotic groups showed significantly higher CP levels did model group. We calculated IRI using IRI = 1.5 + FBG × fasting CP (ng·mL^− 1^ × 333)/2800 and compared IRI at the 6th week among groups. IRI was the highest in model group and the lowest in normal group. IRI in metformin (5.25 ± 1.25 vs 8.21 ± 1.24, *P* < 0.001), and low (6.42 ± 1.20 vs 8.21 ± 1.24, *P* < 0.05) and high dose (6.29 ± 1.01 vs 8.21 ± 1.24, *P* < 0.05) probiotic groups was significantly lower than in model group.

**Table 2:** Effect of CPCM on oral glucose tolerance test, glycosylated hemoglobin, C-peptide and insulin resistance index in *db/db* mice

	Normal (n=10)	Model (n=9)	Metoformin (n=10)	Low dose (n=10)	High dose (n=10)
0min (mmol/L)	6.69±0.71	25.88±6.19^***^	13.10±3.86^###^	19.12±5.87	18.03±4.46^#^
30min (mmol/L)	15.70±2.50	39.11±4.34^***^	31.51±6.57^#^	37.23±4.00	35.58±5.07
60min (mmol/L)	11.98±1.86	36.19±8.55^***^	24.53±4.49^#^	31.39±6.93	27.47±4.12^#^
120min (mmol/L)	7.65±2.35	26.59±6.52^***^	16.19±4.52^##^	19.41±3. 77^#^	18.15±4.49^#^
AUC	1308.81±76.55	3780.06±671.58^***^	2703.04±431.49^#^	3314.28±544.60	3152.50±371.34
glycosylated hemoglobin (ng/ml)	7.78±1.99	17.27±1.01^***^	12.33±1.44^###^	15.74±1.27^#^	15.28±2.04^#^
C-peptide (mmol/L)	2.51±0.36	2.19±0.16^*^	2.53±0.45^#^	2.40±0.19^#^	2.52±0.31^#^
insulin resistance index	3.44±0.22	8.21±1.24^***^	5.25±1.25^##^	6.42±1.20^#^	6.29±1.01^#^

^*^P <0.05, ^***^P <0.001 vs normal group; ^#^ *P* <0.05, ^##^ *P* <0.01, and ^*###*^ *P* <0.001 vs model group

### The effects of the CPCM on lipid metabolism, liver weight and liver index in *db/db* mice

**Fig. 3:**
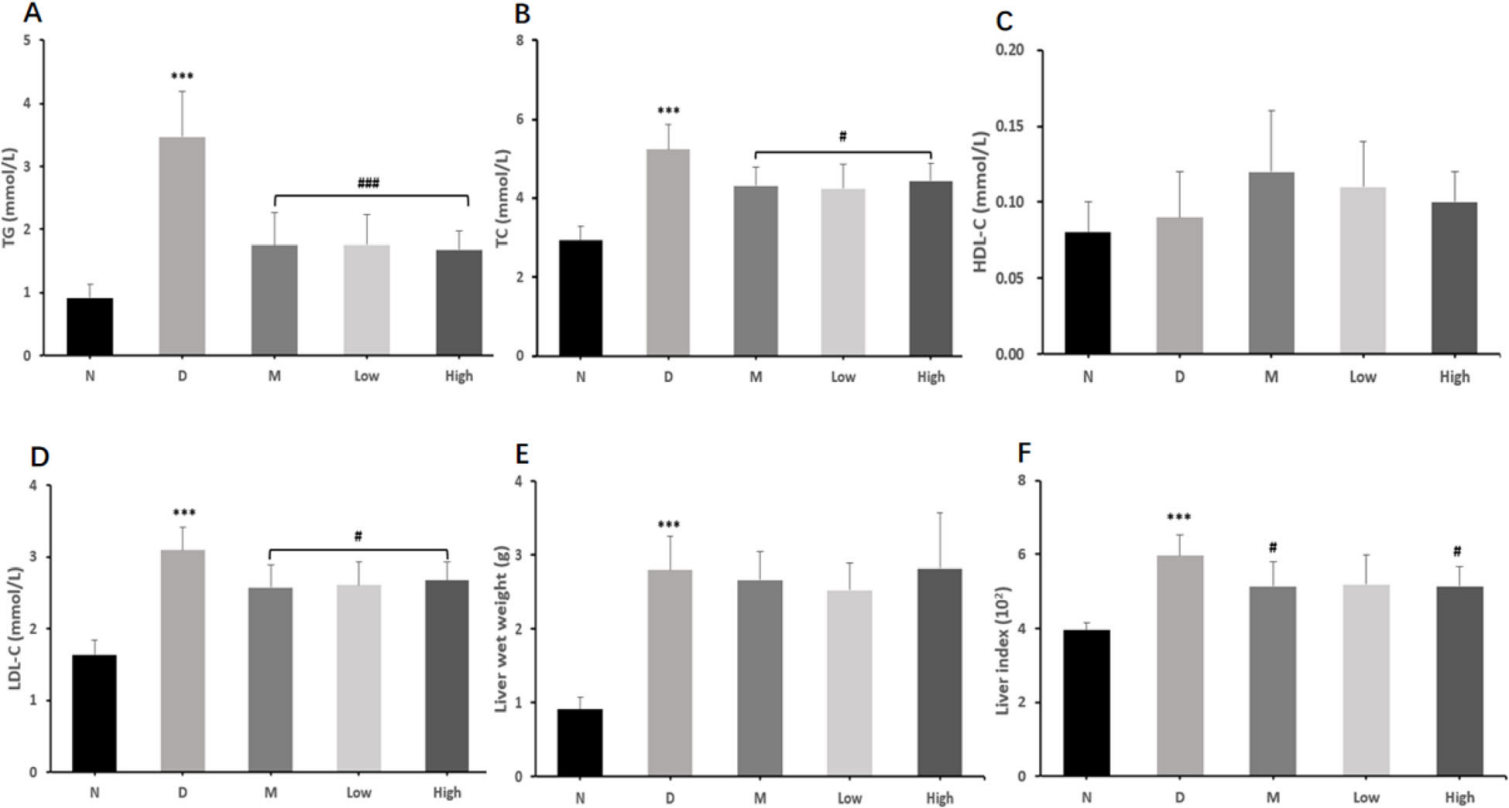
Effects of CPCM on lipid metabolism, liver weight and liver index in *db/db* mice. ^***^ *P* < 0.001 vs normal group; ^#^ *P* < 0.05 and ^###^ *P* < 0.001 vs model group. **a**. Triglyceride (mmol/L); **b**. Total cholesterol (mmol/L); **c**. High density lipoprotein-cholesterol (mmol/L); **d**. Low density lipoprotein-cholesterol (mmol/L); **e**. Liver wet weight (g); **f**. Liver index (10^2^)

As in Table 3 and Fig. 3, the effects of the CPCM on lipid metabolism, liver weight and liver index in *db/db* mice were assessed at the 6th week. Model group showed the highest levels of triglycerides (TG), total cholesterol (TC), low-density lipoprotein cholesterol (LDL-C) and high density lipoprotein cholesterol (HDL-C), compared with normal group (*P* < 0.001). Metformin (1.75 ± 0.52 vs 3.47 ± 0.73 mmol/L, *P* < 0.001), and low (1.76 ± 0.48 vs 3.47 ± 0.73 mmol/L, *P* < 0.001) and high dose (1.68 ± 0.30 vs 3.47 ± 0.73 mmol/L, *P* < 0.001) probiotic groups showed significantly lower levels of TG at the 6th week. Metformin (4.31 ± 0.48 vs 5.24 ± 0.63 mmol/L, *P* < 0.05), and low (4.24 ± 0.62 vs 5.24 ± 0.63 mmol/L, *P* < 0.05) and high dose (4.44 ± 0.44 vs 5.24 ± 0.63 mmol/L, *P* < 0.05) probiotic groups showed significantly lower levels of TC at the 6th week. LDL-C levels also showed similar trend. At the 6th week, HDL-C levels were the highest in metformin group (0.12 ± 0.04 mmol/L), followed by low (0.11 ± 0.03 mmol/L) and high dose (0.10 ± 0.02 mmol/L) probiotic groups but without statistical significance, compared with model group. Compared with the normal group, model group showed significantly higher liver index (5.97 ± 0.56 vs 3.96 ± 0.21, *P* < 0.01) at 6th week of experiment. At the 6th week, metformin (5.15 ± 0.66 vs 5.97 ± 0.56, *P* < 0.05) and high dose probiotic (5.14 ± 0.53 vs 5.97 ± 0.56, *P* < 0.05) groups showed significantly lower liver index.

**Table 3:** Effects of CPCM on lipid metabolism, liver weight and liver index in *db/db* mice

	Normal (n=10)	Model (n=9)	Metoformin (n=10)	Low dose (n=10)	High dose (n=10)
Triglyceride (mmol/L)	0.92±0.21	3.47±0.73^***^	1.75±0.52^###^	1.76±0.48^###^	1.68±0.30^###^
Total cholesterol (mmol/L)	2.92±0.36	5.24±0.63^***^	4.31±0.48^#^	4.24±0.62^#^	4.44±0.44^#^
High density lipoprotein-cholesterol (mmol/L)	0.08±0.02	0.09±0.03	0.12±0.04	0.11±0.03	0.10±0.02
Low density lipoprotein-cholesterol (mmol/L)	1.63±0.22	3.10±0.32^***^	2.58±0.31^#^	2.61±0.32^#^	2.68±0.25^#^
Liver wet weight (g)	0.91±0.16	2.80±0.46^***^	2.67±0.38	2.53±0.37	2.82±0.76
Liver index (10^2^)	3.96±0.21	5.97±0.56^***^	5.15±0.66^#^	5.20±0.78	5.14±0.53^#^

^***^P <0.001 vs normal group; ^#^ *P* <0.05 and ^###^ *P* <0.001 vs model group

### The effects of CPCM on renal weight and index, 24 h microalbuminuria (24 h malb), urine ketone and urine glucose in *db/db* mice

**Fig. 4:**
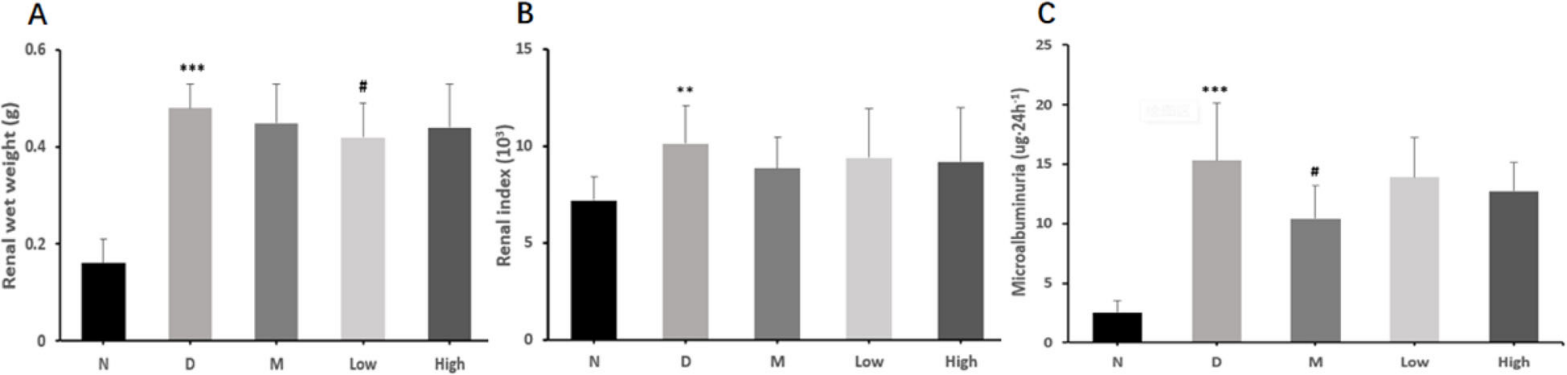
Effect of CPCM on renal weight and index and 24 h microalbuminuria in *db/db* mice. ^**^ *P* < 0.01, ^***^ *P* < 0.001vs normal group; ^#^ *P* < 0.05 vs model group. **a**. Renal wet weight (g); **b**. Renal index (10^3^); **c**.24 h microalbuminuria (ug·24 h^− 1^)

As given in Table 4 and Fig. 4, the effects of CPCM on renal weight and index, 24 h microalbuminuria (24 h malb), urine ketone and urine glucose in *db/db* mice were assessed at the 6th week as well. At the 6th week, model group had the highest renal index, with statistical significance compared with normal group (10.13 ± 1.97 vs 7.18 ± 1.23, *P* < 0.01). Low and high dose probiotic groups showed lower renal index than model group, but not significant. At the 6th week, model group had significantly higher 24 h malb than did normal group (15.33 ± 4.83 vs 2.57 ± 0.96μg/24 h, *P* < 0.001); metformin, and low and high dose probiotic groups showed lower 24 h malb than did model group, but only with statistical significance between metformin and model group (10.44 ± 2.79 vs 15.33 ± 4.83μg/24 h, *P* < 0.05). At the 6th week, metformin, and low and high dose probiotic groups showed slightly lower levels of urinary ketone and glucose than model group.

**Table 4:** Effect of CPCM on renal weight and index, 24h microalbuminuria, urinary ketone and urine glucose in *db/db* mice

	Normal (n=10)	Model (n=9)	Metoformin (n=10)	Low dose (n=10)	High dose (n=10)
Renal wet weight (g)	0.16±0.05	0.48±0.05^***^	0.45±0.08	0.42±0.07^#^	0.44±0.09
Renal index (10^3^)	7.18±1.23	10.13±1.97^**^	8.83±1.60	9.40±2.55	9.19±2.77
24hour microalbuminuria (ug·24h^-1^)	2.57±0.96	15.33±4.83^***^	10.44±2.79^#^	13.91±3.31	12.73±2.42
Urinary ketone (mmol/l)	0 (0, 0)	0.25 (0,3.9)^*^	0 (0,0.125)	0 (0,0.75)	0 (0,0.5)
Urine glucose (mmol/l)	0 (0, 0)	55 (24.5,55)^***^	21 (14,28)	28 (24.5,55)	28 (14,55)

^*^P <0.05, ^**^ *P* <0.01 and ^***^ *P* <0.001 vs normal group; ^#^ *P* <0.05 vs model group

### The starting copy number of *Escherichia* and *Bifidobacterium* in *db/db* mice

**Fig. 5:**
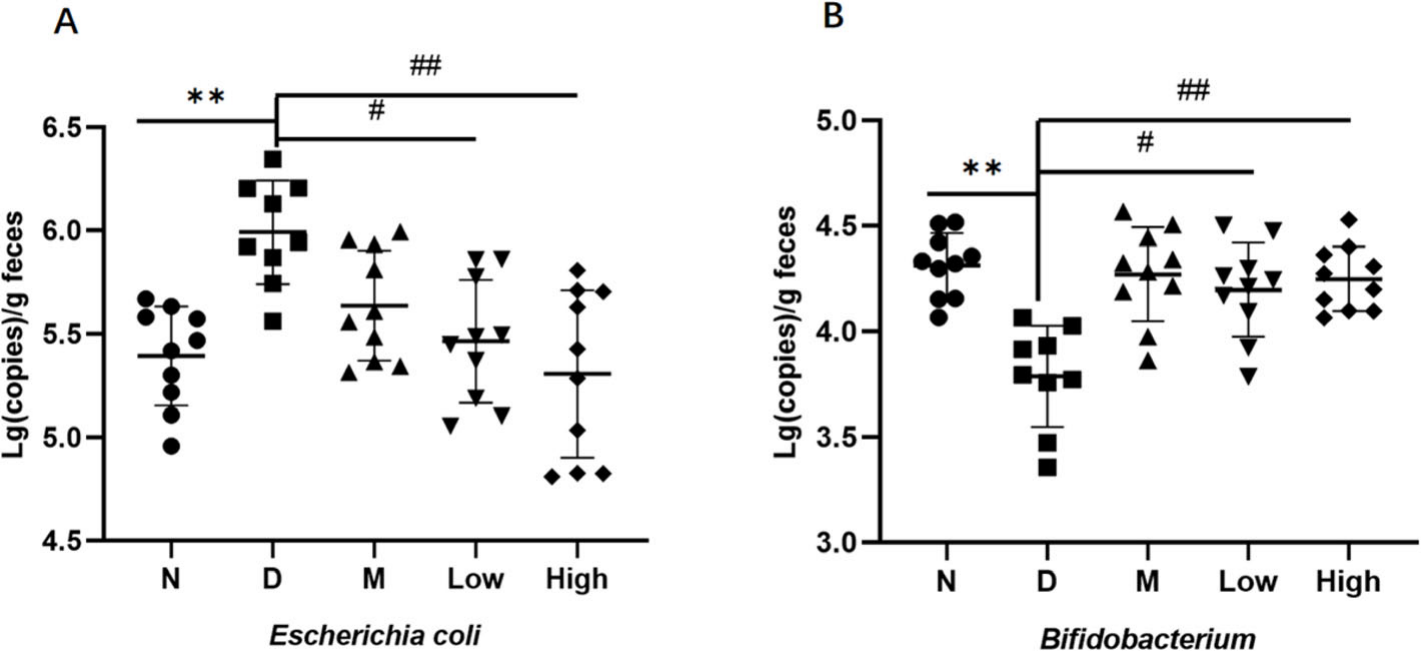
The starting copy number of *Escherichia* and *Bifidobacterium* in *db/db* mice. ^**^ *P* < 0.01 vs normal group; ^#^ *P* < 0.05, ^##^ *P* < 0.01 vs model group. **a**. *Escherichia;* **b**. *Bifidobacterium*

Table 5 and Fig. 5 showed the starting copy number of *Escherichia* and *Bifidobacterium* in different groups. At the 6th week, the starting copy number of *Bifidobacterium* was significantly lower in the gut of model group than in normal group (3.78 ± 0.37 vs 4.31 ± 0.14, *P* < 0.01). At the 6th week, metformin (4.28 ± 0.29 vs 3.78 ± 0.37, *P* < 0.05), and low (4.21 ± 0.24 vs 3.78 ± 0.37, *P* < 0.05) and high dose probiotic (4.26 ± 0.17 vs 3.78 ± 0.37, *P* < 0.05) groups showed significantly higher starting copy number of *Bifidobacterium*. At the 6th week, the starting copy number of *Escherichia* was significantly higher in model group than in normal group (5.99 ± 0.27 vs 5.38 ± 0.34, *P* < 0.01); the lowest in high dose probiotic group (5.31 ± 0.41 vs 5.99 ± 0.27, *P* < 0.01), followed by low dose group (5.50 ± 0.41 vs 5.99 ± 0.27, *P* < 0.05) and meformin group (5.65 ± 0.33 vs 5.99 ± 0.27, *P* < 0.05) with statistical significance compared with model group. The probiotics showed dose-dependent effects on *Escherichia* and *Bifidobacterium* in the gut in *db/db* mice.

**Table 5:** The starting copy number of *Escherichia* and *Bifidobacterium* in *db/db* mice

Groups	n	*Escherichia*	*Bifidobacterium*
Normal	10	5.38±0.34	4.31±0.14
Model	9	5.99±0.27^**^	3.78±0.37^**^
Metformin	10	5.65±0.33^#^	4.28±0.29^#^
Low dose	10	5.50±0.41^#^	4.21±0.24^#^
High dose	10	5.31±0.41^##^	4.26±0.17^##^

^**^P <0.01 vs normal group; ^#^ *P* <0.05, ^##^ *P* <0.01 vs model group

### Histological analysis of the effects of CPCM on pancreas and liver in *db/db* mice

**Fig. 6:**
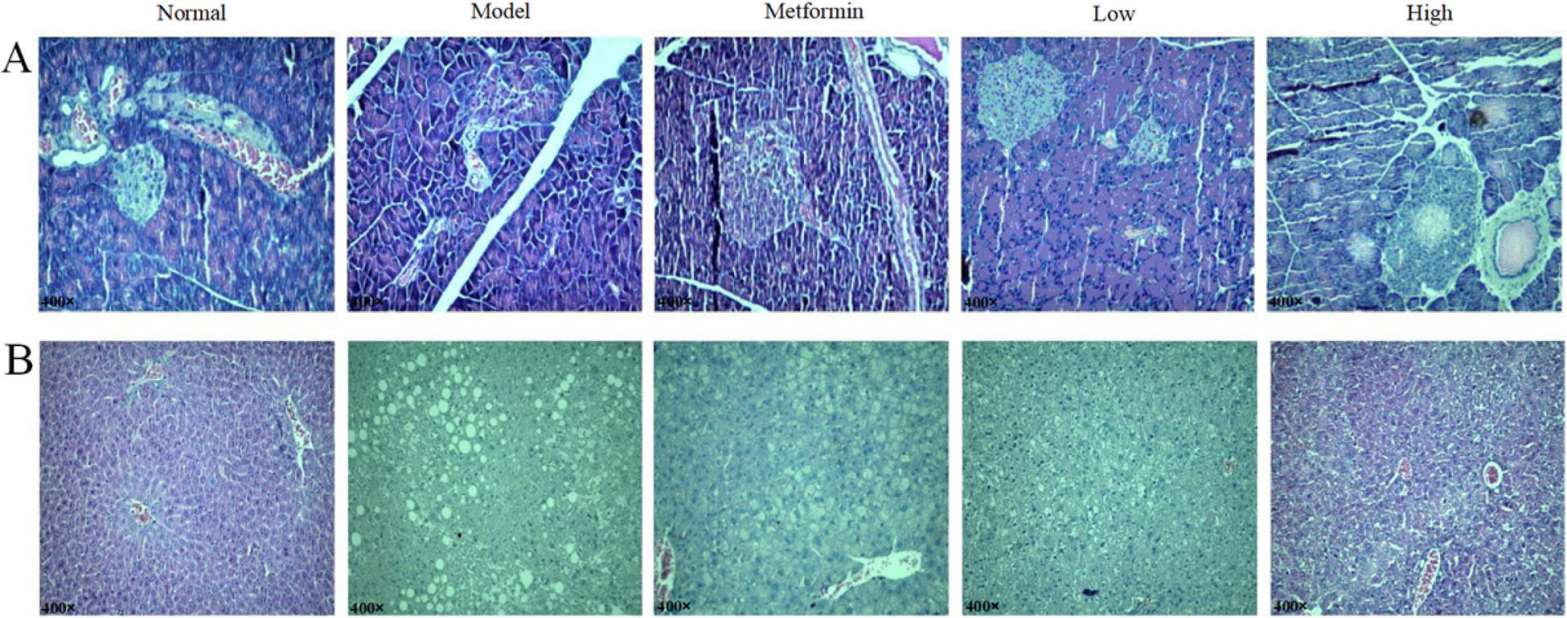
Histological analyses of the effects of CPCM on pancreas and liver in *db/db* mice. Hematoxylin-eosin (HE) stained pancreatic(**a**) microsections(original magnification 400×) and hepatic(**b**) microsections (original magnification 400×) (*n* = 4 images/group)

As shown in Fig. 6a, the histological analysis of the effects of CPCM on pancreas and liver in *db/db* mice were evaluated at the 6th week of experiment. Pancreatic tissue from *db/db* mice of each group was stained with HE method. Pancreatic tissue of normal group was of normal structure, with round and quasi-circular pancreas islet, normal numbered and well-defined. Compared with normal group, model group showed islet with irregular form, reduced number, with cells swollen and vacuoles, hemangiectasis and hyperaemia. Compared with model group, metformin, low and high dose probiotic groups showed islet with larger form, increased cell number. As in Fig. 6b, the histological analysis of the effects of CPCM on the liver in *db/db* mice were also assessed. Liver tissue from *db/db* mice of each group was stained with HE method. Normal group showed well-defined liver tissue with normal structure. Compared with normal group, model group showed liver tissue with severe fatty, swollen and some ballooning changes. Compared with model group, metformin, low and high dose probiotic group showed liver tissue with less fatty, less swollen and less fat granule changes.

## Discussion

Probiotics have many functions, including anti-oxidation, anti-cancer, anti-inflammation, and improved metabolism and immunological function [29–31]. An increasing number of studies have shown that probiotics have enormous potential in treating metabolic diseases, such as diabetes and obesity [31, 32]. Etiology and pathophysiology of T2DM is not still clear and is a topic of research. Current research holds the viewpoint that *db/db* mice are the best choice for the animal models of T2DM and diabetic nephropathy [33]. Effects of CPCM on the *db/db* mice: Improvement in disturbed glycemic metabolism:high and low dose probiotics lowered blood glucose in different extents in the study, with the more marked lowering extent in the high dose probiotic group when the blood glucose of diabetic group was still going up. Meanwhile, probiotics also exerted some roles by decreasing OGTT and HbA1c and increasing CP of d*b/db* mice. Therefore, it is reasonable to believe that composite probiotics can improve glycemic metabolism in T2DM mice in short term assessed by FBG and OGTT and in long term assessed by HbA1c [34]. Nonetheless, the decrease in HbA1c is not marked and significantly higher than that of normal controls, which is possibly explained by the short-term intervention (6 weeks) using probiotic milk. Indeed, normalization of HbA1c takes even more time. Concentrations of CP in the circulation reflect the function of islet cells and are a vital indicator in the diagnosis and treatment of T2DM [35]. In the current study, the high and low dose probiotic groups showed significantly higher CP and lower IRI than did the model group, indicating improvement in insulin resistance in *db/db* mice. Pathological section from pancreas of *db/db* mice showed that the possible mechanism is the inhibiting effects of probiotics on the autoimmnity destruction to β-cell. Improvement of lipid metabolism: Probiotics contain anti-oxident roles and can regulate lipid metabolism [36–38], as evidenced by Yadav [39] and colleagues. In their study, 8-weeks treatment with probiotic dahi containing *Lactobacillus acidophilus* and *Lactobacillus casei* of high fructose fed-induced insulin resistant rats, compared with controls, not only reduced fructose in the liver, but also reduced TC, LDL-C and VLDL levels and thus improved the insulin resistance. In consistency with their study, 6 weeks treatment with low and high-dose probiotic milk significantly lowered the serum TG, TC and LDL-C, with the marked reduction of TG in *db/db* mice in the current study. Effects of CPCM on renal function of the *db/db* mice: Diabetic nephropathy is the most severe chronic complication of DM, caused by its sub-optimal management [40]. 24 h malb is the most sensistive biomarker for early phase diabetic nephropathy and lowering the 24 h malb is an important prognostic indicator in treatment of nephropathy [41]. In fact, *db/db* mice can be characterized by increased 24 h malb after 7 weeks of birth and nephropathy can be the main cause of their mortality. In the current study, 24 h urinary samples were collected after 6 weeks in all groups and 24 h malb was significantly higher OGTT in *db/db* mice than in normal control group. Metformin and low and high dose probiotic groups showed significantly lower 24 h malb levels than did model group, and it was the lowest in metformin group. The results also showed that probiotics can reduce the ketonuria and urine glucose levels in *db/db* mice. It can be speculated from the pathophysiology of diabetic nephropathy that probiotics may improve the renal function by regulating the lipid metabolism and improving the anti-oxident reactions [42]. Effects of CPCM on gut microbiota in *db/db* mice: Considerable research has focused on the relationship between gut microbiota alteration and T2DM and current findings hold the viewpoint that changes in gut microbiota may exert roles in development of T2DM. Turnbaugh et al. reported that gut microbiota not only plays vital parts in energy acquision but also in maintaining and improving the balance of the immune system [43], since most of immune cells exist in the gut and are in close relationship with the gut microbiota [44–49]. Gut microbiota of high fat-induced T2DM mice at the early phase translocates into the inflammatory tissues such as adipose tissue and blood system and adhesion of symbiotic bacteria on intestinal mucosa [50], which provided evidence and basis for treatment of obesity-related T2DM by regulating gut microbiota [51]. In addition, Zhang and colleagues found that berberine, a main component of coptis chinensis traditional medicine lowers the LPS and inflammatory mediators through regulating the gut microbiota in animal models with insulin resistance and obesity [52]. Moreover, some products of gut microbiota, such as insulin-similar substance from *Escherichia coli* result in development and progression of T2DM [53]. In the current study, low and high dose probiotics after 6 weeks decreased the number of *Escherichia coli*, increased that of Bifidobacterium, and showed a marked reduction of *Escherichia coli* in the low dose group and marked increase in Bifidobacterium in the high dose group. The number of Bifdobacterium improves the intestinal barrier function and shows a negative relationship with LPS levels [54, 55].

## Conclusion

In conclusion, high-dose CPCM reduces the FBG, OGTT, HbA1c and IRI, increases CP levels in *db/db* mice and improves the pancreas. In addition, composite probiotics regulates the lipid metabolism, and improves fatty liver, renal function, and gut microbiota, which may be related to various probiotics acting through protecting the function of islets and regulating intestinal flora disturbance.

## Supplementary Information

**Additional file 1: Table S1**. The starting copy number of 4 lactic acid bacteria.

**Additional file 2: Table S2**. Primer sequence for probiotics in the current study

**Additional file 3: Figure S1**. The genome DNA electrophoresis of intestinal microbial in *db/db* mice

**Additional file 4: Figure S2**. The detection Figure of *Lactobacillus kefiranofaciens* by RT-qPCR. A Standard curve; B Amplification curves; C Melting curve

**Additional file 5: Figure S3**. The detection Figure of *Lactobacillus plantarum* by RT-qPCR. A Standard curve; B Amplification curves; C Melting curve

**Additional file 6: Figure S4**. The detection Figure of *Lactobacillus helveticus* by RT-qPCR. A Standard curve; B Amplification curves; C Melting curve

**Additional file 7: Figure S5**. The detection Figure of *Lactococcus lactis* by RT-qPCR. A Standard curve; B Amplification curves; C Melting curve

**Additional file 8: Figure S6**. The detection Figure of *Escherichia* by RT-qPCR. A Standard curve; B Amplification curves; C Melting curve

**Additional file 9: Figure S7**. The detection Figure of *Bifidobacterium* by RT-qPCR A Standard curve; B Amplification curves; C Melting curve.

## Data Availability

The datasets analysed during the current study are available from the corresponding author(Prof. Xin-Hua Nabi) on reasonable request.

## Abbreviations

24 h malb: 24 h urinary microalbumin
BW: Body weight
CICC: China Center of Industrial Culture Collection
CP: C-peptide
CPCM: Composite probiotics camel milk
DM: Diabetes mellitus
FBG: Fasting blood glucose
GCG: Proglucagon
GLP-1: Glucagon-like peptide-1
GPR43/41: G protein-coupled receptor 43/41
HbAlc: Glycated hemoglobin
HDL-C: High density lipoprotein cholesterol
IL: Interleukin
IRI: Insulin resistance index
LDL-C: Low-density lipoprotein cholesterol
OGTT: Oral glucose tolerance test
PC1/3: Proconvertase 1/3
T2DM: Type 2 diabetes mellitus
TC: Total cholesterol
TFCW: Traditional fermented cheese whey
TG: Triglycerides

## References

[1] Lakerveld J, Bot SDM, Chinapaw MJ, van Tulder MW, van Oppen P, Dekker JM, Nijpels G (2008). Primary prevention of diabetes mellitus type 2 and cardiovascular diseases using a cognitive behavior program aimed at lifestyle changes in people at risk: design of a randomized controlled trial. BMC Endocr Disord.

[2] Xu Y, Wang L, He J, Bi Y, Li M, Wang T, Wang L, Jiang Y, Dai M, Lu J, Xu M, Li Y, Hu N, Li J, Mi S, Chen CS, Li G, Mu Y, Zhao J, Kong L, Chen J, Lai S, Wang W, Zhao W, Ning G, 2010 China Noncommunicable Disease Surveillance Group (2013). Prevalence and control of diabetes in Chinese adults. JAMA..

[3] International Diabetes Federation. IDF Diabetes Atlas. 2019. https://www.idf.org/aboutdiabetes/what-is-diabetes/facts-figures.html. Accessed 20 Dec 2020.

[4] Herring R, Jones RH, Russell-Jones DL (2014). Hepatoselectivity and the evolution of insulin. Diabetes Obes Metab.

[5] Yan W, Yang X, Zheng Y, Ge D, Zhang Y, Shan Z, Simu H, Sukerobai M, Wang R (2005). The metabolic syndrome in Uygur and Kazak populations. Diabetes Care.

[6] Wang L, Tao Y, Xie Z (2010). Prevalence of metabolic syndrome, insulin resistance, impaired fasting blood glucose, and dyslipidemia in Uygur and Kazak populations. J Clin Hypertens (Greenwich).

[7] Li N, Wang H, Yan Z (2012). Ethnic disparities in the clustering of risk factors for cardiovascular disease among the Kazakh, Uygur, Mongolian and Han populations of Xinjiang: a cross-sectional study. BMC Public Health.

[8] Zhang HW, Jiang S, Xu YC (2013). A cross-sectional study on serum uric acid level and the distribution of metabolic syndrome among Uigur, Han and Kazak prediabetic groups in Xinjiang. Chin J Epidemiol.

[9] Yan WL, Li XS, Wang Q, Huang YD, Zhang WG, Zhai XH, Wang CC, Lee JH (2015). Overweight, high blood pressure and impaired fasting glucose in Uyghur, Han, and Kazakh Chinese children and adolescents. Ethn Health.

[10] NaBi XH, Rehemu N, Luo L (2007). Effects of traditional fermented cheese whey on experimental atherosclerosis in rats. Chin J New Drugs.

[11] NaBi XH, Ma CY, Manaer T (2016). Anti-atherosclerotic effect of traditional fermented cheese whey in atherosclerotic rabbits and identification of probiotics. BMC Complement Altern Med.

[12] Wang JM, Zhao RG, Xiao DG (2005). Primary Study on Lactobacillus casei Starter Powder with High Activity. J Univ Sci Technol tianjin.

[13] Nagata S, Asahara T, Ohta T, Yamada T, Kondo S, Bian L, Wang C, Yamashiro Y, Nomoto K (2011). Effect of the continuous intake of probiotic-fermented milk containing Lactobacillus casei strain Shirota on fever in a mass outbreak of norovirus gastroenteritis and the faecal microflora in a health service facility for the aged. Br J Nutr.

[14] Musso G, Gambino R, Cassader M (2010). Obesity, diabetes, and gut microbiota the hygiene hypothesis expanded?. Diabetes Care.

[15] Larsen N, Vogensen FK, van den Berg FW (2010). Gut microbiota in human adults with type 2 diabetes differs from non-diabetic adults. PLoS One.

[16] Wang M, Bi J (2007). Medium optimization for kefiran synthesis by Lactobacillus kefiranofaciens. Indus Microbiol.

[17] Zhou J, Zheng M (2009). Malleable protein matrix and contact dermatitis. Inter J Dermatol Venereol.

[18] Wang J (2005). Progress of physiological function of lactic acid bacteria. Food Ferment Sichuan.

[19] Liu SM, Man CX, Li L, Jiang YJ (2013). Research on Immunomodulatory of lactic acid Bacteria. Chin J Food Nutr.

[20] Liu AG, Liu YL, Wang ZJ (2008). Molecular identification of wild wine-related yeasts isolated from spontaneous wine fermentation in Ningxia district. J Northwest Univ (Natural Science Edition).

[21] Wang ZJ, Liu YL, Liu AG (2008). Survey on yeast population dynamics during wine spontaneous fermentation in Xinjiang. J Agr Univ Huazhong.

[22] Latipa A, Xue T, Xin-Hua N (2014). Molecular biological identification of one lactic acid bacteria in Xinjiang traditional fermented camel milk. J Med Univ Xinjiang.

[23] Manaer T, Yu L, Zhang Y, Xiao XJ, Nabi XH (2015). Anti-diabetic effects of shubat in type 2 diabetic rats induced by combination of high-glucose-fat diet and low-dose streptozotocin. J Ethnopharmacol.

[24] Dinareer D, Lu L, Jialehasibieke S (2018). Probiotic characteristics of the lactic acid bacteria and yeasts in Xinjiang-traditional fermented dairy products. China Microecology.

[25] Jialehasibieke S, Xin S, Amanguli J (2019). Probiotic characteristics of probiotics in Xinjiang traditional fermented dairy products. China Microecology J.

[26] Wang Y, Dinareer D, Wu Y (2020). Composite probiotics alleviate type 2 diabetes by regulating intestinal microbiota and inducing GLP-1 secretion in db/db mice. Biomed Pharmacother.

[27] Hendijani F, Akbari V (2018). Probiotic supplementation for management of cardiovascular risk factors in adults with type II diabetes: a systematic review and metaanalysis. Clin Nutr.

[28] Teng Y, Li D, Guruvaiah P, Xu N, Xie Z (2018). Dietary supplement of large yellow tea ameliorates metabolic syndrome and attenuates hepatic Steatosis in db/db mice. Nutrients..

[29] Yoo JY, Kim SS (2016). Probiotics and prebiotics: present status and future perspectives on metabolic disorders. Nutrients..

[30] Singh S, Sharma RK, Malhotra S, Pothuraju R, Shandilya UK (2017). Lactobacillus rhamnosus NCDC17 ameliorates type-2 diabetes by improving gut function, oxidative stress and inflammation in high-fat-diet fed and streptozotocintreated rats. Benef Microbes.

[31] Payne AN, Chassard C, Zimmermann M (2011). The metabolic activity of gut microbiota in obese children is increased compared with normal-weight children and exhibits more exhaustive substrate utilization. Nutr Diabetes.

[32] Mekkes MC, Weenen TC, Brummer RJ (2014). The development of probiotic treatment in obesity: a review. Benef Microbes.

[33] Allen TJ, Cooper ME, Lan HY (2004). Use of genetic mouse models in the study of diabetic nephropathy. Curr Diab Rep.

[34] Chen WX (2011). Standardization of glycated hemoglobin measurement. Chin J Diabetes.

[35] Le TK, Hosaka T, Nguyen TT (2015). Bifidobacterium species lower serum glucose, increase expressions of insulin signaling proteins, and improve adipokine profile in diabetic mice. Biomed Res.

[36] Falcinelli S, Picchietti S, Rodiles A, Cossignani L, Merrifield DL, Taddei AR, Maradonna F, Olivotto I, Gioacchini G, Carnevali O (2015). Lactobacillus rhamnosus lowers zebrafish lipid content by changing gut microbiota and host transcription of genes involved in lipid metabolism. Sci Rep.

[37] Mohammadi Sartang M, Mazloomi SM, Tanideh N (2015). The effects of probiotic soymilk fortified with Omega-3 on blood glucose, lipid profile, Haematological and oxidative stress, and inflammatory parameters in Streptozotocin Nicotinamide-induced diabetic rats. J Diabetes Res.

[38] Ebrahimi ZS, Nasli-Esfahani E, Nadjarzade A (2017). Effect of symbiotic supplementation on glycemic control, lipid profiles and microalbuminuria in patients with non-obese type 2 diabetes: a randomized, double-blind, clinical trial. J Diabetes Metab Disord.

[39] Yadav H, Jain S, Sinha PR (2007). Antidiabetic effect of probiotic dahi containing Lactobacillus acidophilus and Lactobacillus casei in high fructose fed rats. Nutrition..

[40] Bo H, Huang SM, Wu WH (2011). Status quo of diabetic nephropathy and countermeasures. Chin J Nephrol..

[41] Xiao L, Sun L, Liu FY (2010). New progress in the formation of proteinuria in diabetic kidney disease. Chin J Nephrol.

[42] Tesch GH, Lim AK (2011). Recent insights into diabetic renal injury from the db/db mouse model of type 2 diabetic nephropathy. Am J Physiol Renal Physiol.

[43] Turnbaugh PJ, Ley RE, Mahowald MA, Magrini V, Mardis ER, Gordon JI (2006). An obesity-associated gut microbiome with increased capacity for energy harvest. Nature..

[44] Jia W, Li H, Zhao L, Nicholson JK (2008). Gut microbiota: a potential new territory for drug targeting. Nat Rev Drug Discov.

[45] Gomes AC, Bueno AA, de Souza RG (2014). Gut microbiota, probiotics and diabetes. Nutr J.

[46] Everard A, Matamoros S, Geurts L (2014). Saccharomyces boulardii administration changes gut microbiota and reduces hepatic steatosis, low-grade inflammation, and fat mass in obese and type 2 diabetic db/db mice. MBio.

[47] Candela M, Biagi E, Soverini M, Consolandi C, Quercia S, Severgnini M, Peano C, Turroni S, Rampelli S, Pozzilli P, Pianesi M, Fallucca F, Brigidi P (2016). Modulation of gut microbiota dysbioses in type 2 diabetic patients by macrobiotic Ma-pi 2 diet. Br J Nutr.

[48] Chen F, Wen Q, Jiang J, Li HL, Tan YF, Li YH, Zeng NK (2016). Could the gut microbiota reconcile the oral bioavailability conundrum of traditional herbs?. J Ethnopharmacol.

[49] Bordalo Tonucci L, Dos Santos KM, De Luces Fortes Ferreira CL (2017). Gut microbiota and probiotics: focus on diabetes mellitus. Crit Rev Food Sci Nutr.

[50] Amar J, Serino M, Lange C (2011). Involvement of tissue bacteria in the onset of diabetes in humans: evidence for a concept. Diabetologia..

[51] Nova E, Pérez de Heredia F, Gómez-Martínez S (2016). The role of probiotics on the microbiota: effect on obesity. Nutr Clin Pract.

[52] Zhang X, Zhao Y, Zhang M, Pang X, Xu J, Kang C, Li M, Zhang C, Zhang Z, Zhang Y, Li X, Ning G, Zhao L (2012). Structural changes of gut microbiota during berberine-mediated prevention of obesity and insulin resistance in high-fat diet-fed rats. PLoS One.

[53] Tanaka J, Fukuda Y, Shintani S, Hori K, Tomita T, Ohkusa T, Matsumoto T, Miwa H (2005). Influence of antimicrobial treatment for helicobacter pylori infection on the intestinal microflora in Japanese macaques. J Med Microbiol.

[54] Griffiths EA, Duffy LC, Schanbacher FL, Qiao H, Dryja D, Leavens A, Rossman J, Rich G, Dirienzo D, Ogra PL (2004). In vivo effects of bifidobacteria and lactoferrin on gut endotoxin concentration and mucosal immunity in Balb/c mice. Dig Dis Sci.

[55] Wang ZT, Yao YM, Xiao GX, Sheng ZY (2004). Risk factors of development of gut-derived bacterial translocation in thermally injured rats. World J Gastroenterol.

