# Supplementary material for: The beneficial effects of the composite probiotics from camel milk on glucose and lipid metabolism, liver and renal function and gut microbiota in db/db mice
Source: BMC Complement Med Ther. 2021 Apr 22;21:127. doi: 10.1186/s12906-021-03303-4 (PMC8061000; doi:10.1186/s12906-021-03303-4)
Supplement: Supplementary file 1 — Additional file 1: Table S1. The starting copy number of 4 lactic acid bacteria. [file 12906_2021_3303_MOESM1_ESM.doc]

| **Table S1. The starting copy number of 4 lactic acid bacteria** | | | | | |
| --- | --- | --- | --- | --- | --- |
| Groups | n | *L.kefianofaciens* | *L.plantarum* | *L.helveticus* | *L.lactis* |
| Low dose | 10 | 4.68±0.21 | 4.16±0.26 | 4.68±0.50 | 4.62±0.38 |
| High dose | 10 | 4.77±0.29 | 4.74±0.13 | 4.71±0.30 | 4.68±0.34 |
