# Supplementary material for: The beneficial effects of the composite probiotics from camel milk on glucose and lipid metabolism, liver and renal function and gut microbiota in db/db mice
Source: BMC Complement Med Ther. 2021 Apr 22;21:127. doi: 10.1186/s12906-021-03303-4 (PMC8061000; doi:10.1186/s12906-021-03303-4)
Supplement: Supplementary file 2 — Additional file 2: Table S2. Primer sequence for probiotics in the current study [file 12906_2021_3303_MOESM2_ESM.doc]

| **Table S2. Primer sequence for probiotics in the current study** | | | |
| --- | --- | --- | --- |
| probiotics | 5'to3' | amplified fragment length (bp) | TM |
| *L.kefianofaciens* | F:TCTACGCATTCCACCGCTAC | 215 | 55.9 |
|  | R:CAACCAGAAAGTCACGGCTAA |  |  |
| *L. lactis* | F:CGGAGTGCTTATTGCGTTAG | 198 | 60 |
|  | R:TGTAGCGGTGAAATGCGTAG |  |  |
| *L.helveticus* | F:GCATTCCACCGCTACACAT | 209 | 55.9 |
|  | R:CAACCAGAAAGTCACGGCTA |  |  |
| *L.plantarum* | F:GCGAACTGGTGAGTAACACG | 124 | 62 |
|  | R:ATAGCCGAAGCCATCTTTCA |  |  |
| *Escherichia* | F:GGAGCAAACAGCATTAGATACCC | 317 | 54 |
|  | R:AACCCAACATTTCACAACACG |  |  |
| [*Bifidobacterium*](../../../../C:/Users/Administrator/AppData/Local/youdao/dict/Application/7.5.2.0/resultui/dict/javascript:%3B) | F: GTCAGCTCGTGTCGTGAG | 245 | 61.5 |
|  | R:GTCGCATCCCGTTGTACC |  |  |
