# Supplementary figures and images for: The beneficial effects of the composite probiotics from camel milk on glucose and lipid metabolism, liver and renal function and gut microbiota in db/db mice
Source: BMC Complement Med Ther. 2021 Apr 22;21:127. doi: 10.1186/s12906-021-03303-4 (PMC8061000; doi:10.1186/s12906-021-03303-4)

**
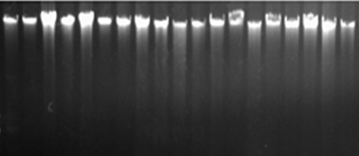
**

Figure S1. The genome DNA electrophoresis of intestinal microbial in *db/db* mice

Supplement: Supplementary file 3 — Additional file 3: Figure S1. The genome DNA electrophoresis of intestinal microbial in db/db mice [file 12906_2021_3303_MOESM3_ESM.docx]
