# Supplementary material for: The beneficial effects of the composite probiotics from camel milk on glucose and lipid metabolism, liver and renal function and gut microbiota in db/db mice
Source: BMC Complement Med Ther. 2021 Apr 22;21:127. doi: 10.1186/s12906-021-03303-4 (PMC8061000; doi:10.1186/s12906-021-03303-4)
Supplement: Supplementary file 6 — Additional file 6: Figure S4. The detection Figure of Lactobacillus helveticus by RT-qPCR. A Standard curve; B Amplification curves; C Melting curve [file 12906_2021_3303_MOESM6_ESM.docx]

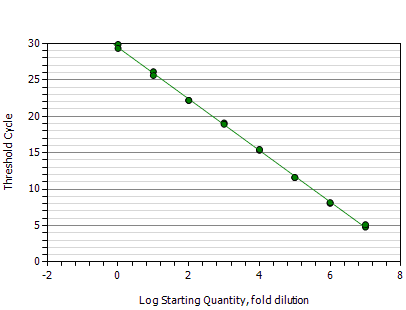

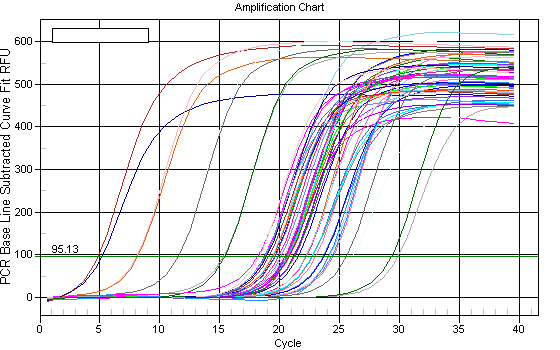


B

A

**
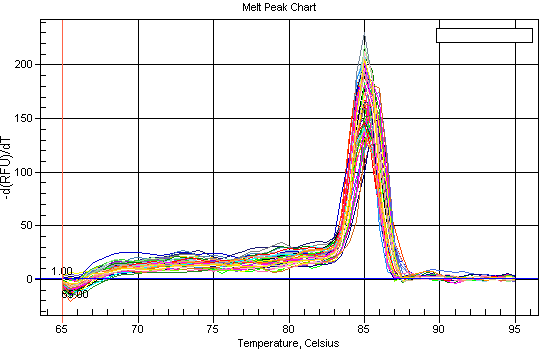
**

C

Figure S4. The detection Figure of *Lactobacillus helveticus* by RT-qPCR

A Standard curve; B Amplification curves; C Melting curve
